# Supplementary material for: Understanding the Role of GLUT2 in Dysglycemia Associated with Fanconi–Bickel Syndrome
Source: Biomedicines. 2022 Aug 29;10(9):2114. doi: 10.3390/biomedicines10092114 (PMC9495670; doi:10.3390/biomedicines10092114)
Supplement: Supplementary file 1 [file biomedicines-10-02114-s001.zip › biomedicines-1844287-supplementary.pdf]

Supplementary Data:

Table S1: List of primers used in this report.

| Sample | Forward Primer | Reverse Primer |
|---|---|---|
| **DNA Sanger Sequencing** | | |
| **Patient 1** | AGCCAAAGTACTTTTTCAACAAACT | AGAGACTGAAGGATGGCTCG |
| **Patient 2** | TCCCCACCTTGATCTCACTC | CAAACATCCCACTCATTCCA |
| **cDNA Sanger sequencing** | | |
| **Patient 1** | 1. ATCGGTGAAATTGCTCCAACCG<br>2. TTTTCATCAGCTGGCCATCG | 1. GGTATCTGGGGCTTTCTGGAC<br>2. AGAGACTGAAGGATGGCTCG |
| **qRT-PCR** | | |
| **GAPDH** | CTGACTTCAACAGCGACACC | TTACTCCTTGGAGGCCATGT |
| **GLUT1** | TGTCGTGTCGCTGTTTGTG | ATGGCCACGATGCTCAGATA |
| **GLUT3** | AGCTCTCTGGGATCAATGCT | GAGTCCTTCTTCCTGCCCTT |

Table S2: List of 123 miRNA specific to the case 1

| | | |
|---|---|---|
| hsa-let-7e-5p | hsa-miR-331-3p | hsa-miR-552-3p |
| hsa-miR-1-5p | hsa-miR-342-3p | hsa-miR-554 |
| hsa-miR-103a-3p | hsa-miR-342-5p | hsa-miR-561-3p |
| hsa-miR-107 | hsa-miR-365b-5p | hsa-miR-562 |
| hsa-miR-1183 | hsa-miR-371b-5p | hsa-miR-564 |
| hsa-miR-1202 | hsa-miR-381-3p | hsa-miR-566 |
| hsa-miR-1204 | hsa-miR-425-5p | hsa-miR-567 |
| hsa-miR-1228-3p | hsa-miR-433-3p | hsa-miR-571 |
| hsa-miR-1246 | hsa-miR-450a-5p | hsa-miR-573 |
| hsa-miR-1248 | hsa-miR-451a | hsa-miR-577 |
| hsa-miR-1249-5p | hsa-miR-486-3p | hsa-miR-579-3p |
| hsa-miR-1250-5p | hsa-miR-499a-3p | hsa-miR-580-3p |
| hsa-miR-1258 | hsa-miR-5001-3p | hsa-miR-589-5p |
| hsa-miR-125a-5p | hsa-miR-503-5p | hsa-miR-595 |
| hsa-miR-125b-5p | hsa-miR-506-3p | hsa-miR-599 |
| hsa-miR-1277-3p | hsa-miR-509-5p | hsa-miR-604 |
| hsa-miR-1285-3p | hsa-miR-516b-5p | hsa-miR-605-5p |
| hsa-miR-129-5p | hsa-miR-517b-3p | hsa-miR-610 |
| hsa-miR-1296-5p | hsa-miR-518b | hsa-miR-614 |
| hsa-miR-1304-3p | hsa-miR-518d-3p | hsa-miR-615-5p |
| hsa-miR-1306-5p | hsa-miR-5196-3p+hsa-miR-6732-3p | hsa-miR-617 |

| | | |
|---|---|---|
| hsa-miR-1322 | hsa-miR-519b-5p+hsa-miR-519c-5p+hsa-miR-523-5p+hsa-miR-518e-5p+hsa-miR-522-5p+hsa-miR-519a-5p | hsa-miR-619-3p |
| hsa-miR-143-3p | hsa-miR-520a-3p | hsa-miR-624-3p |
| hsa-miR-144-3p | hsa-miR-520a-5p | hsa-miR-637 |
| hsa-miR-148a-3p | hsa-miR-520b | hsa-miR-638 |
| hsa-miR-148b-3p | hsa-miR-520c-3p | hsa-miR-641 |
| hsa-miR-154-5p | hsa-miR-520e | hsa-miR-642a-3p |
| hsa-miR-181b-2-3p | hsa-miR-520f-3p | hsa-miR-649 |
| hsa-miR-182-5p | hsa-miR-521 | hsa-miR-6511a-5p |
| hsa-miR-18a-5p | hsa-miR-523-3p | hsa-miR-654-3p |
| hsa-miR-1908-3p | hsa-miR-524-3p | hsa-miR-656-3p |
| hsa-miR-196a-3p | hsa-miR-539-3p | hsa-miR-661 |
| hsa-miR-199a-3p+hsa-miR-199b-3p | hsa-miR-539-5p | hsa-miR-663a |
| hsa-miR-19b-3p | hsa-miR-541-3p | hsa-miR-664b-3p |
| hsa-miR-20a-5p+hsa-miR-20b-5p | hsa-miR-544a | hsa-miR-769-5p |
| hsa-miR-221-5p | hsa-miR-545-3p | hsa-miR-93-5p |
| hsa-miR-23c | hsa-miR-548ad-3p | hsa-miR-99b-5p |
| hsa-miR-24-3p | hsa-miR-548al | |
| hsa-miR-25-3p | hsa-miR-548b-3p | |

| | | |
|---|---|---|
| hsa-miR-29a-3p | hsa-miR-548c-5p+hsa-miR-548o-5p+hsa-miR-548am-5p | |
| hsa-miR-30d-5p | hsa-miR-548h-5p | |
| hsa-miR-3158-3p | hsa-miR-548l | |
| hsa-miR-324-5p | hsa-miR-550a-5p | |

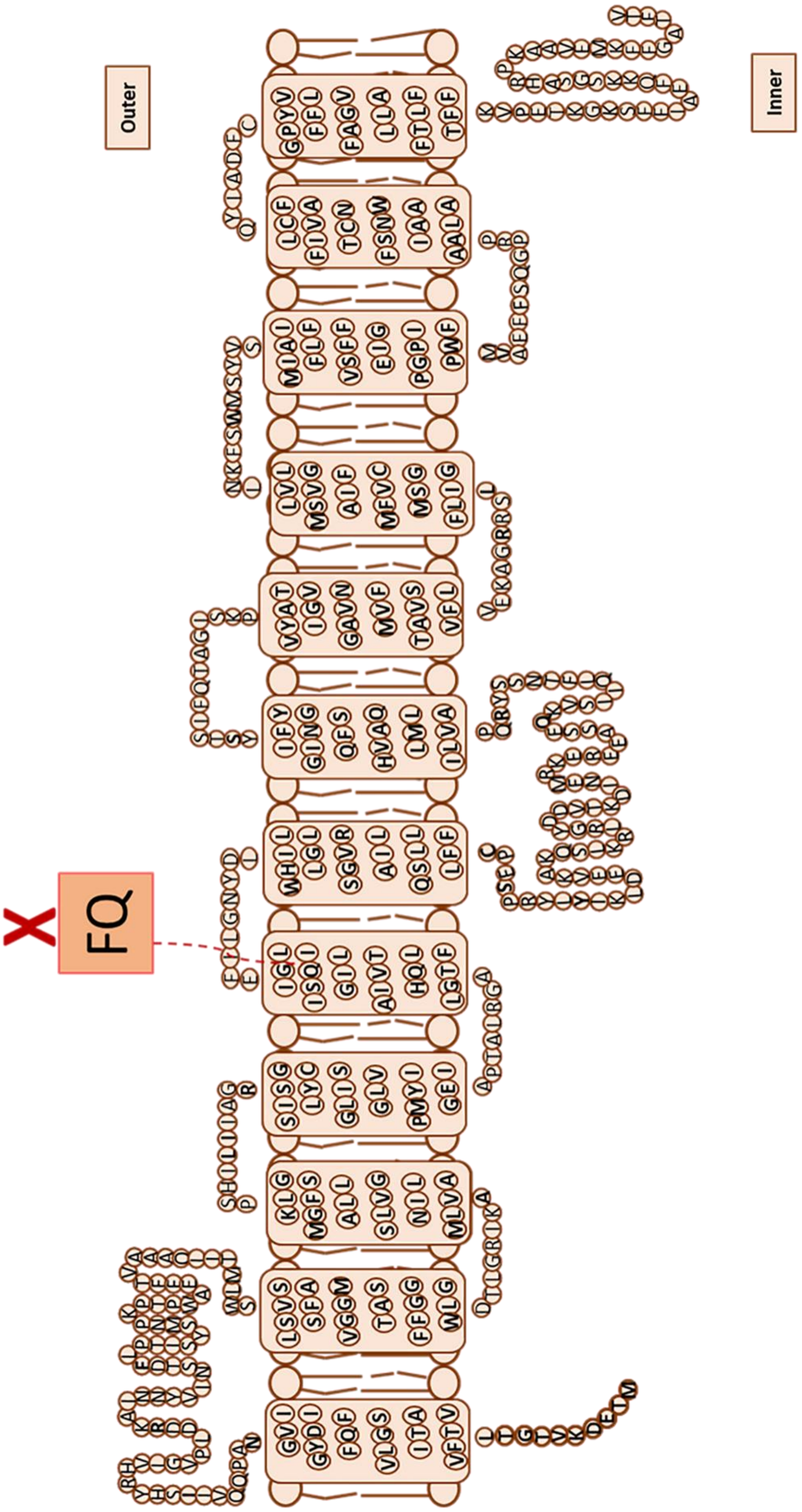


Figure S1: Expected effect of the intronic mutation on GLUT2 topology (no loss or gain of amino acids).

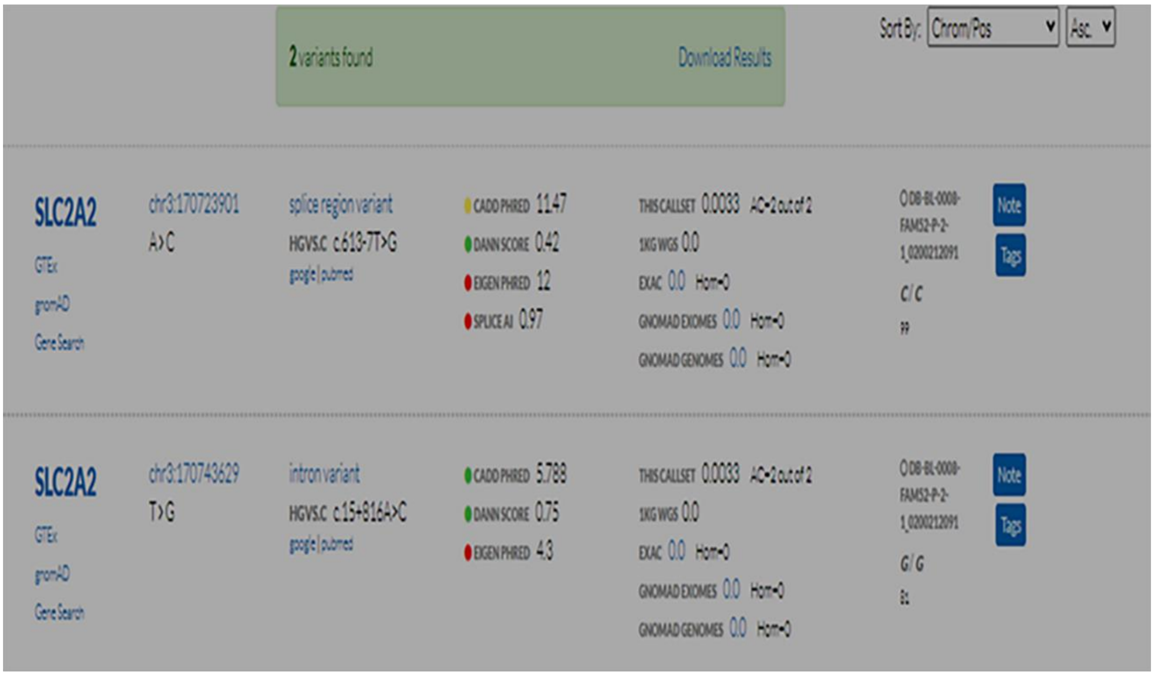


Figure S2: Two variants in *SLC2A2* were found in SeqR. Whole genome sequencing data for case 1 was uploaded in SeqR and two variants (one pathogenic and other likely benign) were detected.

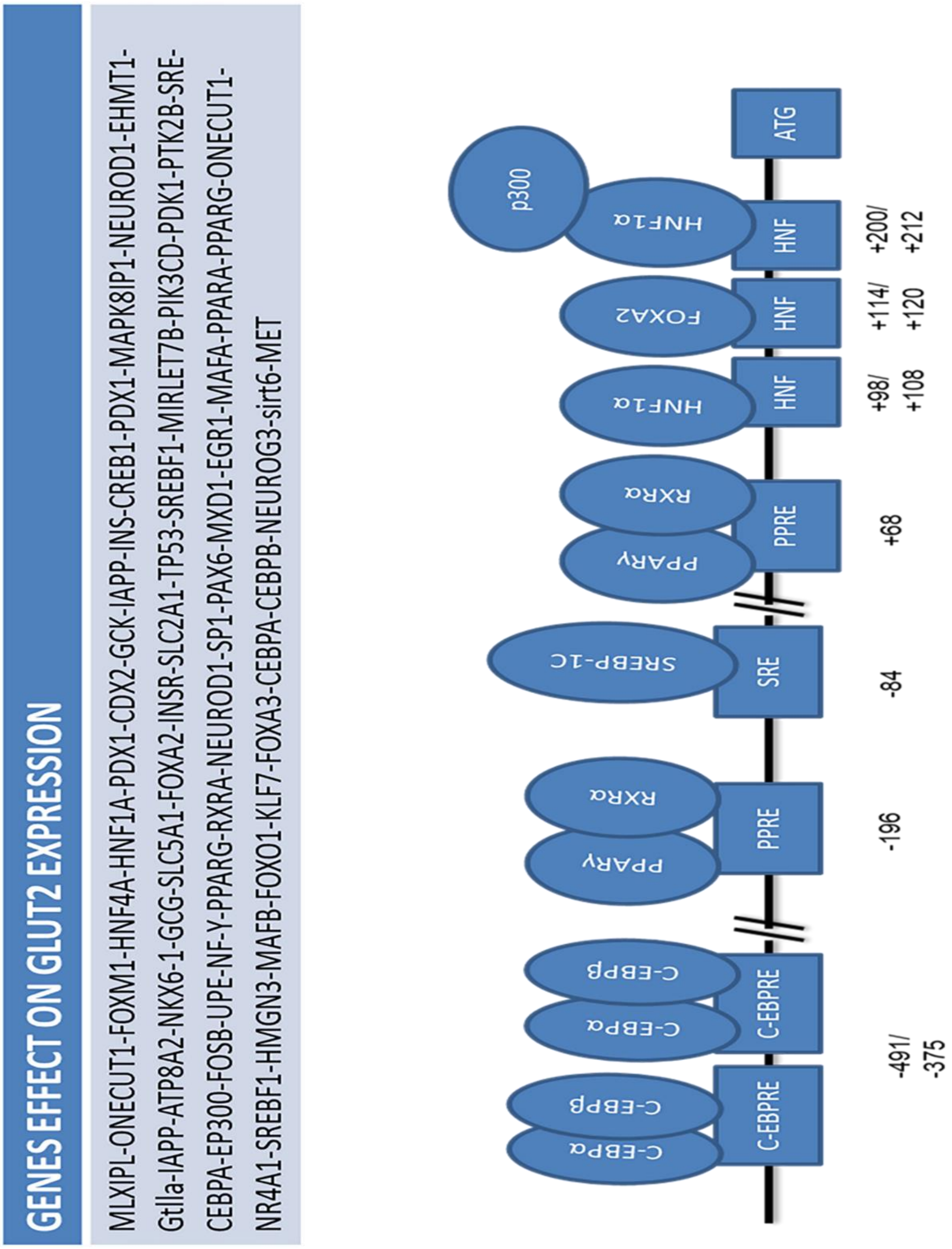


Figure S3: Genes reported to regulate the expression of GLUT2. 60 genes, most of the transcription factors, were found in the literature to regulate the expression of GLUT2 (summarized in upper panel). The relative binding sites within the GLUT2 promoter of a select number of these transcription factors are illustrated in the lower panel.

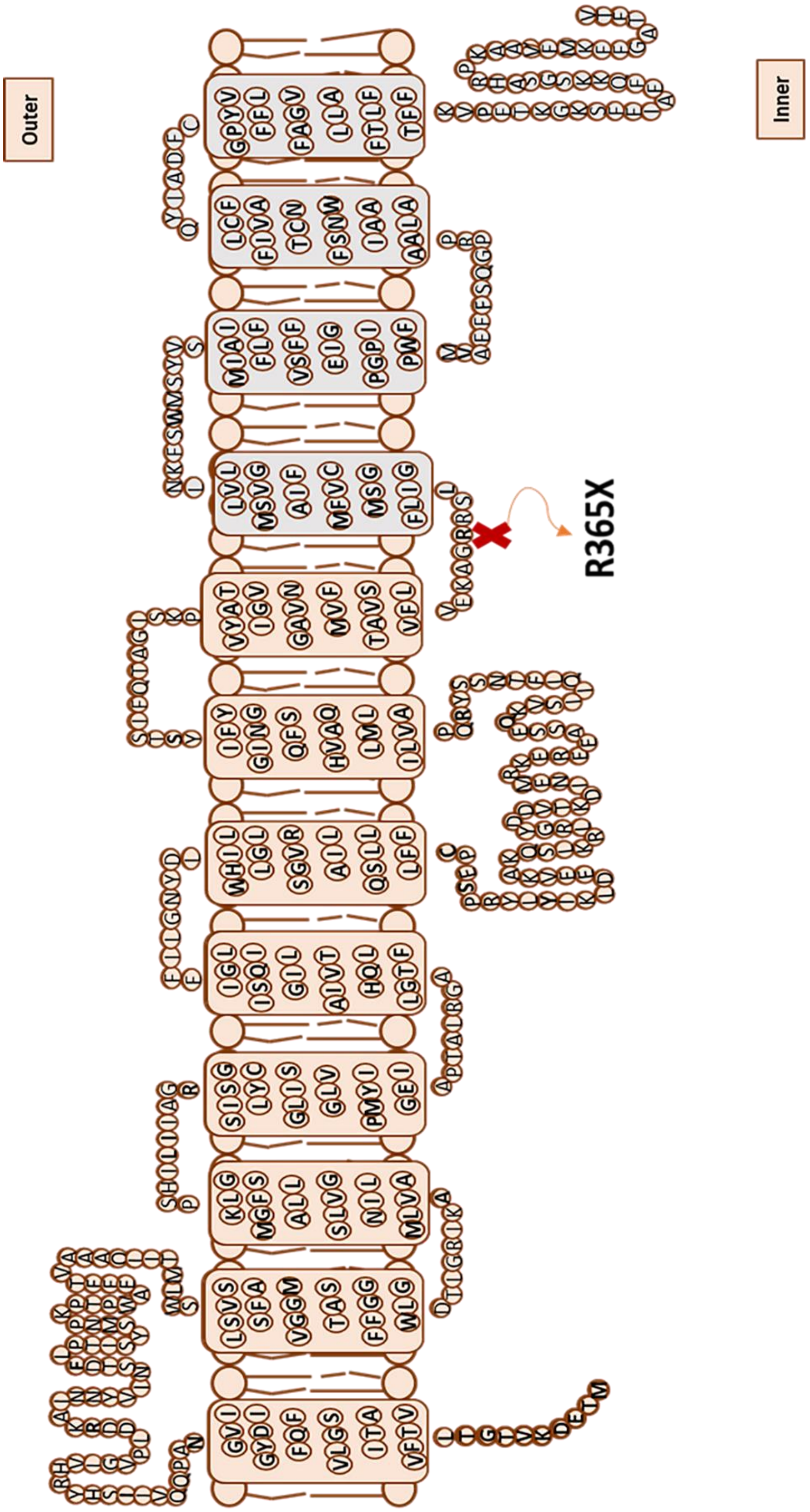


Figure S4: Expected effect of the exonic mutation on GLUT2 topology (truncated protein). The last four transmembrane segments of GLUT2 are expected to be lost in the patient.

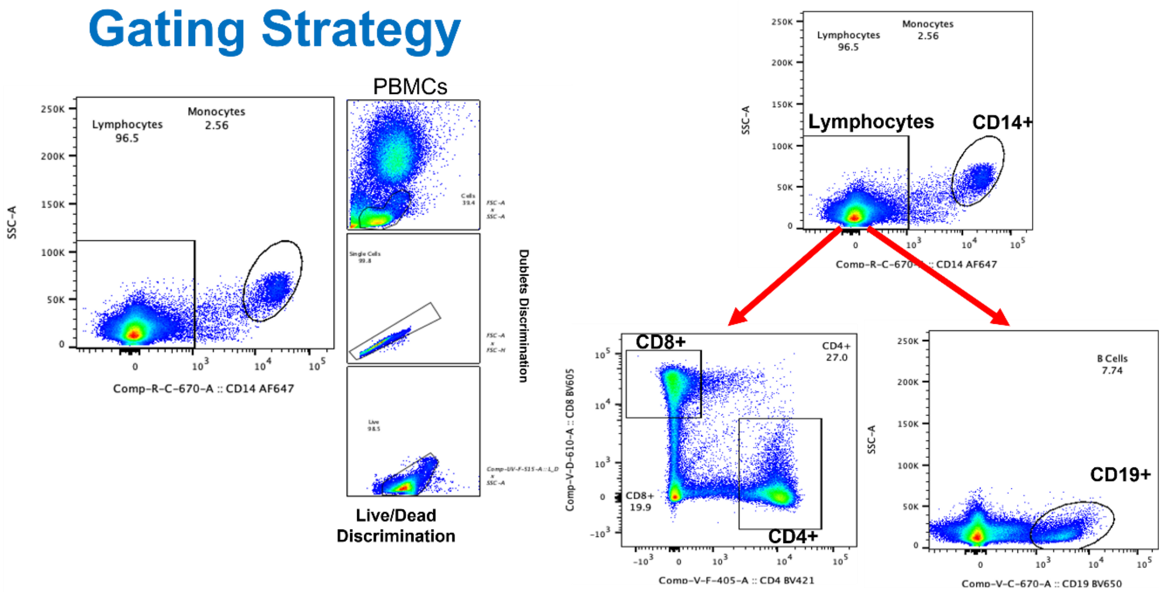


Figure S5: Flow cytometry to assess GLUT2 expression in each cell type in healthy PBMCs. Illustration of the gating strategy used. T Lymphocytes (CD4+, CD8+), B lymphocytes (CD19+), monocytes (CD14+) extracted from a healthy control and the individual cells were sorted using the gate strategy.

a)

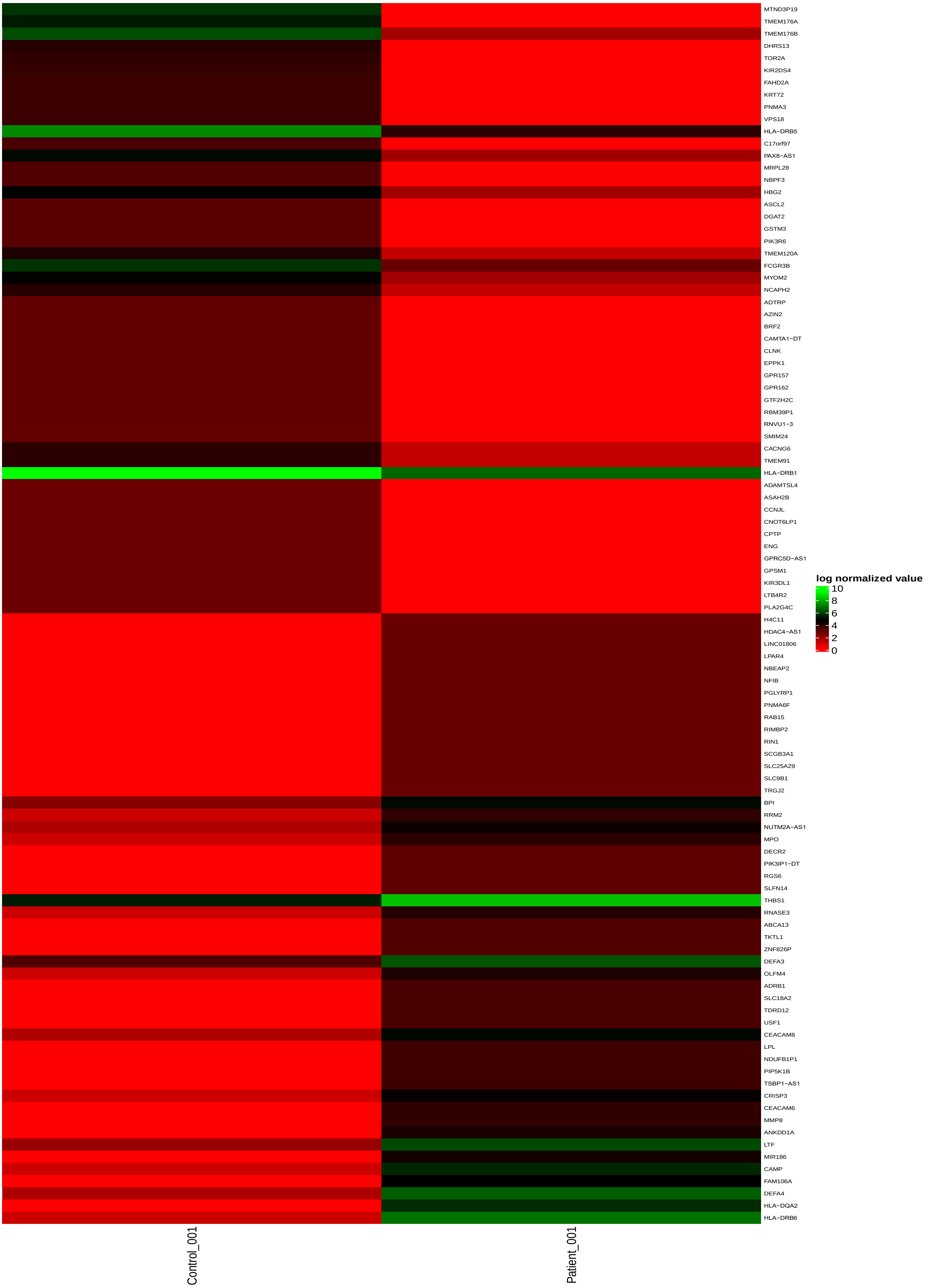
MTND3P19
TMEM176A
TMEM176B
DHRS13
TOR2A
KIR2DS4
FAHD2A
KRT72
PNMA3
VPS18
HLA−DRB5
C17orf97
PAX8−AS1
MRPL28
NBPF3
HBG2
ASCL2
DGAT2
GSTM3
PIK3R6
TMEM120A
FCGR3B
MYOM2
NCAPH2
ADTRP
AZIN2
BRF2
CAMTA1−DT
CLNK
EPPK1
GPR157
GPR162
GTF2H2C
RBM39P1
RNVU1−3
SMIM24
CACNG6
TMEM91
HLA−DRB1
ADAMTSL4
ASAH2B
CCNJL
CNOT6LP1
CPTP
ENG
GPRC5D−AS1
GPSM1
KIR3DL1
LTB4R2
PLA2G4C
H4C11
HDAC4−AS1
LINC01806
LPAR4
NBEAP2
NFIB
PGLYRP1
PNMA6F
RAB15
RIMBP2
RIN1
SCGB3A1
SLC25A29
SLC9B1
TRGJ2
BPI
RRM2
NUTM2A−AS1
MPO
DECR2
PIK3IP1−DT
RGS6
SLFN14
THBS1
RNASE3
ABCA13
TKTL1
ZNF826P
DEFA3
OLFM4
ADRB1
SLC18A2
TDRD12
USF1
CEACAM8
LPL
NDUFB1P1
PIP5K1B
TSBP1−AS1
CRISP3
CEACAM6
MMP8
ANKDD1A
LTF
MIR186
CAMP
FAM106A
DEFA4
HLA−DQA2
HLA−DRB6
log normalized value
10
8
6
4
2
0
Control_001
Patient_001

b)

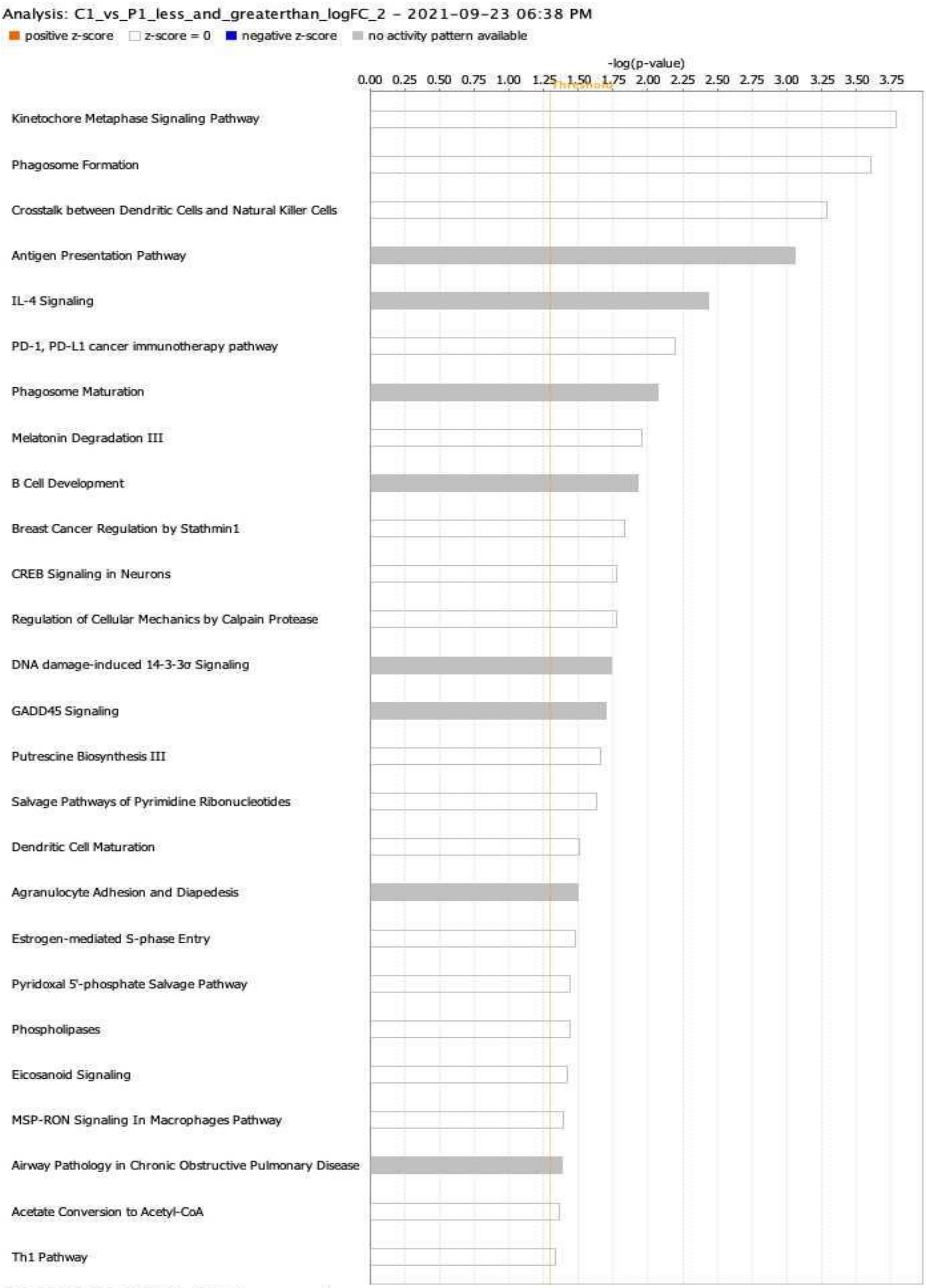


© 2000-2021 QIAGEN. All rights reserved.

c)

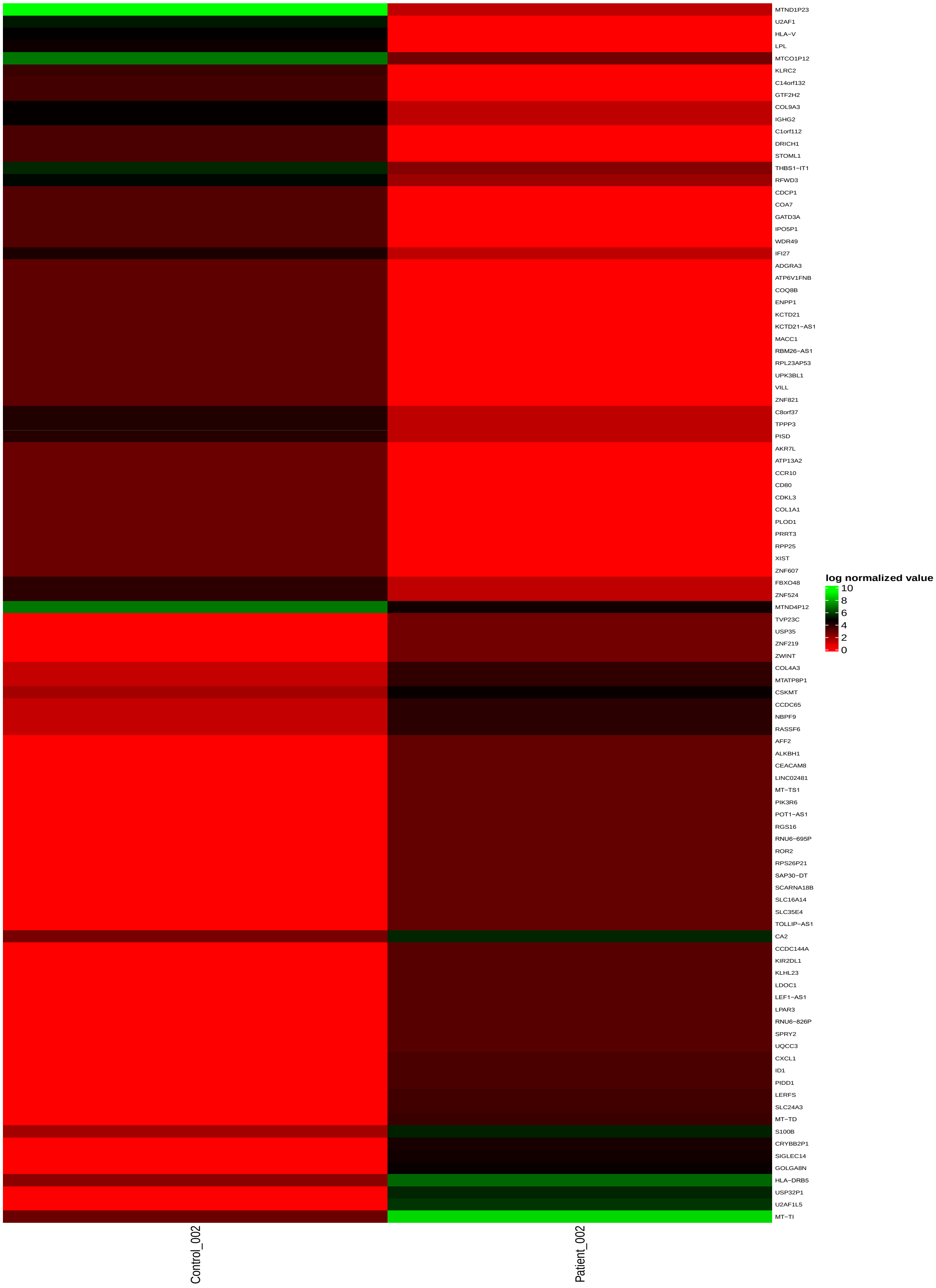
MTND1P23
U2AF1
HLA−V
LPL
MTCO1P12
KLRC2
C14orf132
GTF2H2
COL9A3
IGHG2
C1orf112
DRICH1
STOML1
THBS1−IT1
RFWD3
CDCP1
COA7
GATD3A
IPO5P1
WDR49
IFI27
ADGRA3
ATP6V1FNB
COQ8B
ENPP1
KCTD21
KCTD21−AS1
MACC1
RBM26−AS1
RPL23AP53
UPK3BL1
VILL
ZNF821
C8orf37
TPPP3
PISD
AKR7L
ATP13A2
CCR10
CD80
CDKL3
COL1A1
PLOD1
PRRT3
RPP25
XIST
ZNF607
FBXO48
ZNF524
MTND4P12
TVP23C
USP35
ZNF219
ZWINT
COL4A3
MTATP8P1
CSKMT
CCDC65
NBPF9
RASSF6
AFF2
ALKBH1
CEACAM8
LINC02481
MT−TS1
PIK3R6
POT1−AS1
RGS16
RNU6−695P
ROR2
RPS26P21
SAP30−DT
SCARNA18B
SLC16A14
SLC35E4
TOLLIP−AS1
CA2
CCDC144A
KIR2DL1
KLHL23
LDOC1
LEF1−AS1
LPAR3
RNU6−826P
SPRY2
UQCC3
CXCL1
ID1
PIDD1
LERFS
SLC24A3
MT−TD
S100B
CRYBB2P1
SIGLEC14
GOLGA8N
HLA−DRB5
USP32P1
U2AF1L5
MT−TI
log normalized value
10
8
6
4
2
0
Control_002
Patient_002

d)

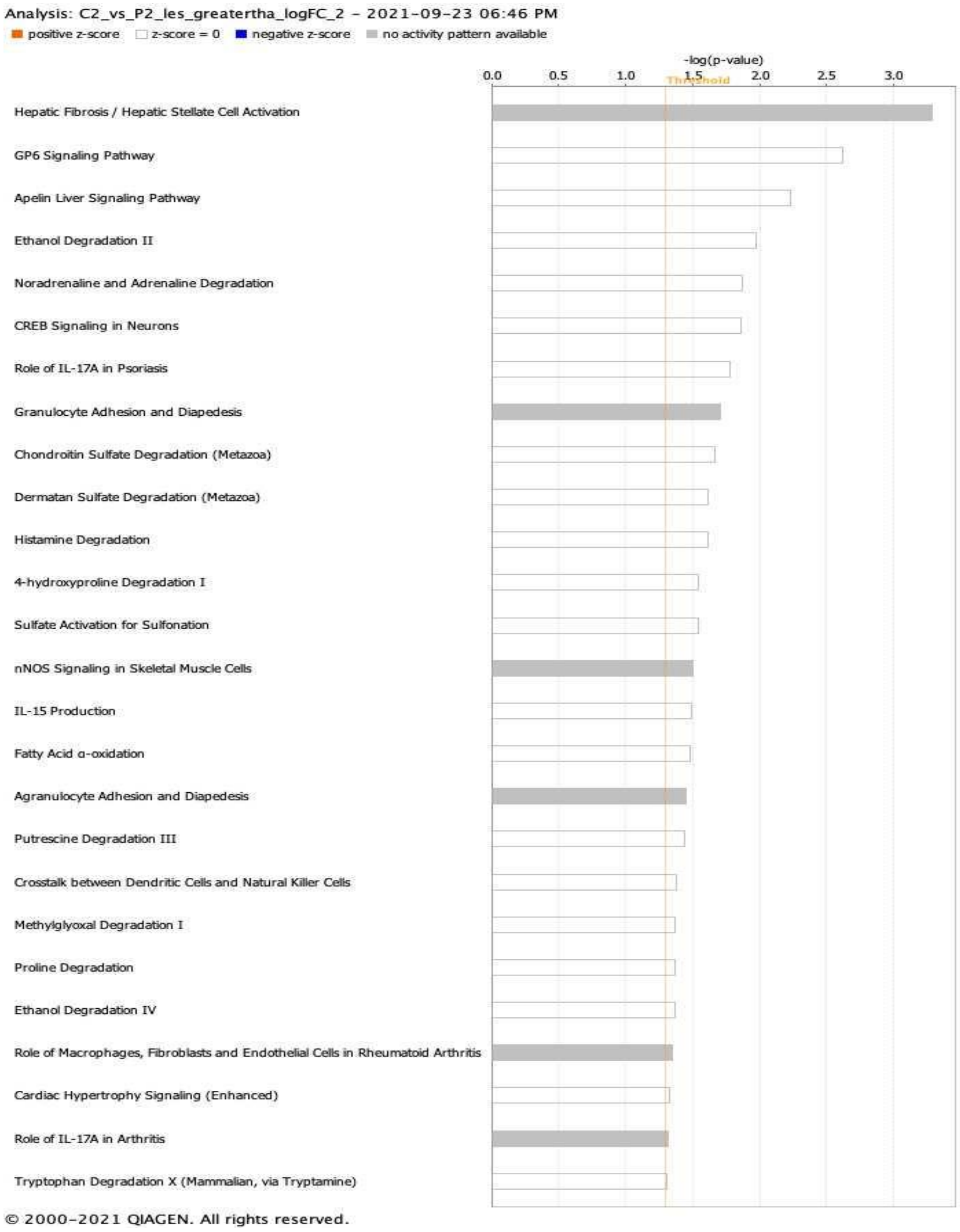


Figure S6: RNA-Seq analysis for patients and their controls. (a & c) Patients 1 and 2 had differentially regulated genes not associated with dysglycemia except for two genes (RETN (resistin) in patient-1 (found in >500 genes differentially regulated list) and ENPP1 in patient-

2) involved in insulin resistance. (b & d) Pathways other than dysglycemia might be dysregulated in patients 1 and 2 in comparison to controls.

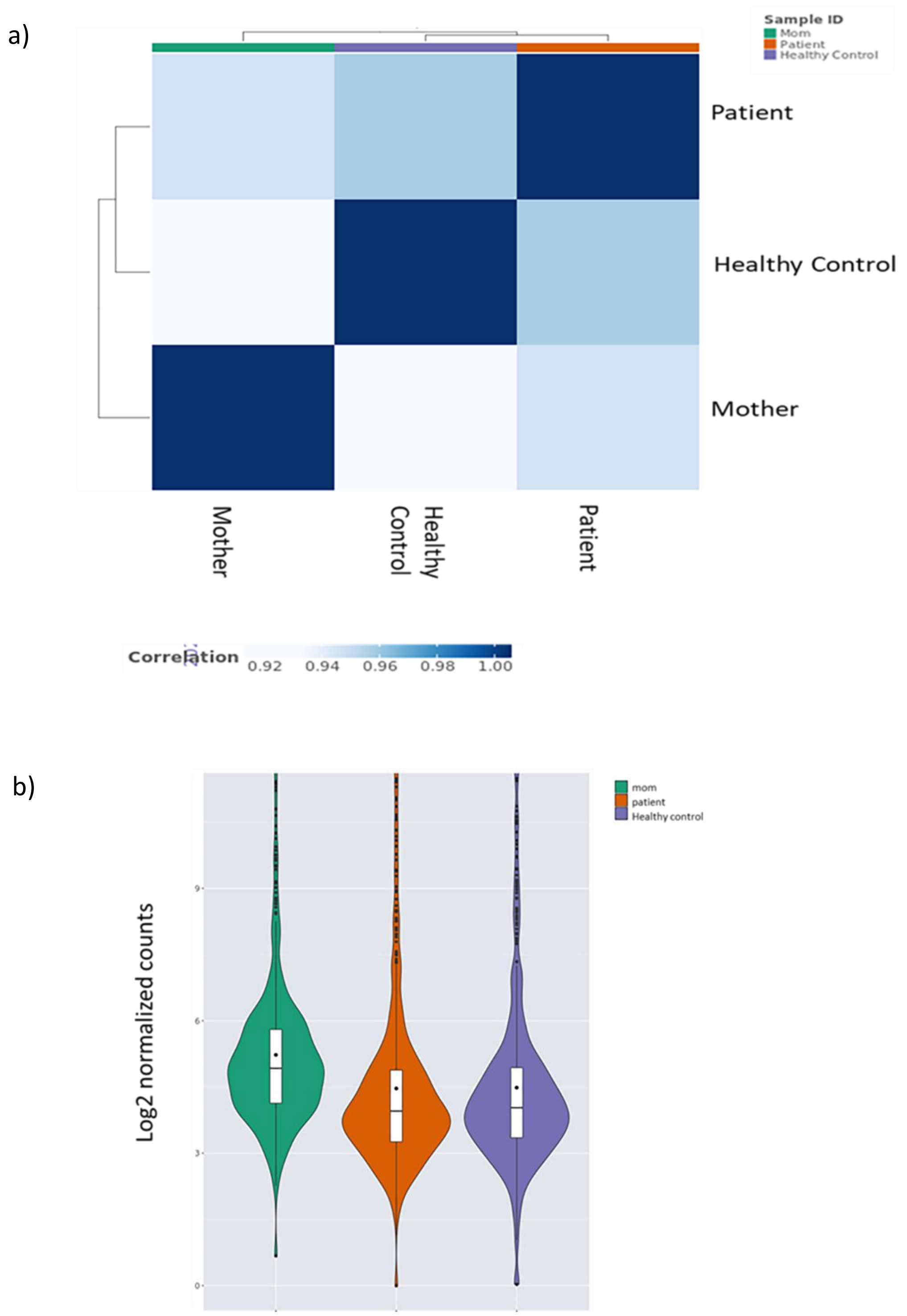


Figure S7: ROSALIND analysis for the data generated from the patient carrying the intronic mutation, the patient's mother, and a healthy control individual. (a) A sample correlation heatmap provides a graphical representation of data, in which the individual values contained in a matrix are represented as colors. In this case, the data matrix contains correlation values between samples, with the darkest blue representing the

strongest correlation. Patient and healthy control showed a strong correlation. (b) a box plot is overlaid on a violin plot to display the distribution of the log of the un-normalized gene counts for each sample. Patient and healthy control showed a more similar data distribution.

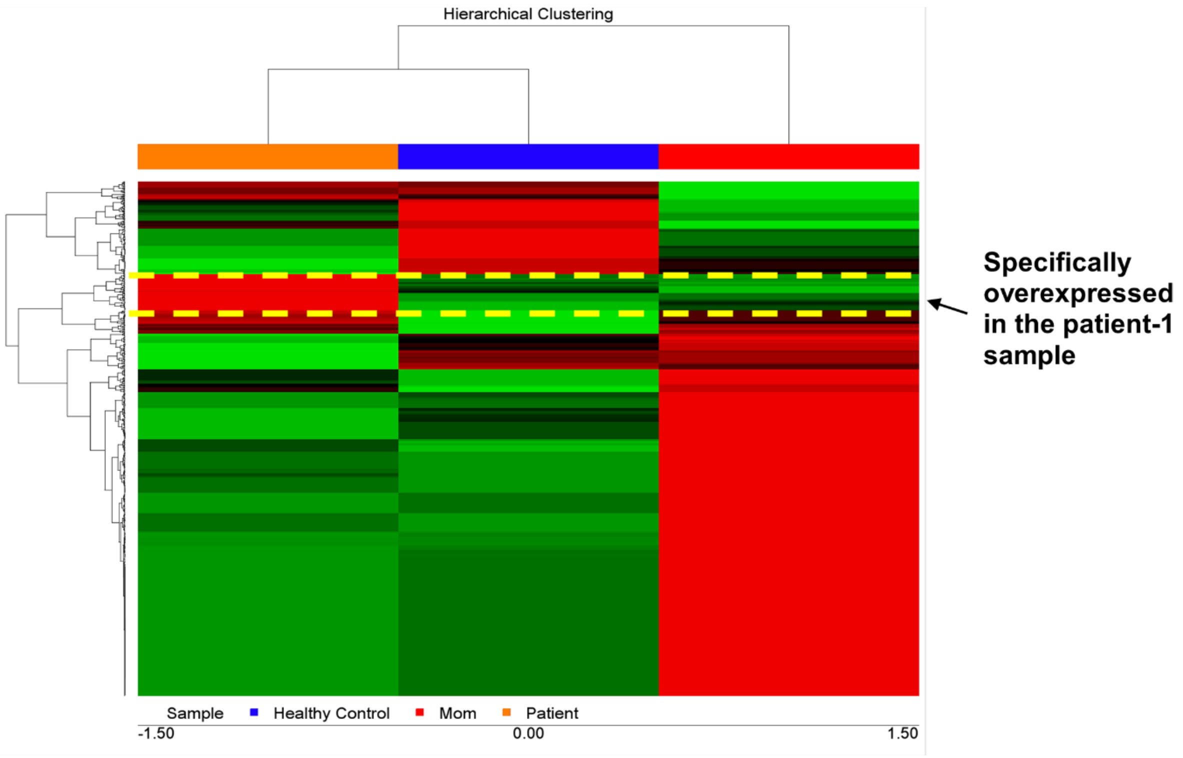


Figure S8. Heat map generated by Partek for case 1. 123 miRNAs were specific to the patient-1, with 118 mapped following IPA analysis. The region marked with the yellow dotted line includes miRNAs specially overexpressed in the patient.

### Diseases and Disorders

| Name | p-value range | # Molecules |
|---|---|---|
| Organismal Injury and Abnormalities | 4.93E-02 - 1.25E-34 | 61 |
| Reproductive System Disease | 3.72E-02 - 1.25E-34 | 38 |
| Inflammatory Disease | 1.40E-02 - 2.96E-22 | 28 |
| Inflammatory Response | 2.80E-02 - 2.96E-22 | 26 |
| Renal and Urological Disease | 4.56E-03 - 2.96E-22 | 19 |

### Molecular and Cellular Functions

| Name | p-value range | # Molecules |
|---|---|---|
| Cellular Development | 4.32E-02 - 3.74E-08 | 31 |
| Cellular Growth and Proliferation | 4.32E-02 - 3.74E-08 | 32 |
| Cell Cycle | 4.62E-02 - 4.43E-05 | 10 |
| Cellular Movement | 3.93E-02 - 8.31E-05 | 18 |
| Free Radical Scavenging | 1.14E-03 - 1.14E-03 | 2 |

### Physiological System Development and Function

| Name | p-value range | # Molecules |
|---|---|---|
| Organismal Development | 4.98E-02 - 7.68E-11 | 17 |
| Embryonic Development | 4.32E-02 - 4.43E-05 | 4 |
| Cardiovascular System Development and Function | 2.46E-02 - 8.61E-05 | 11 |
| Organ Morphology | 1.88E-02 - 8.61E-05 | 9 |
| Hematological System Development and Function | 4.32E-02 - 9.73E-05 | 9 |

**Network 1:**

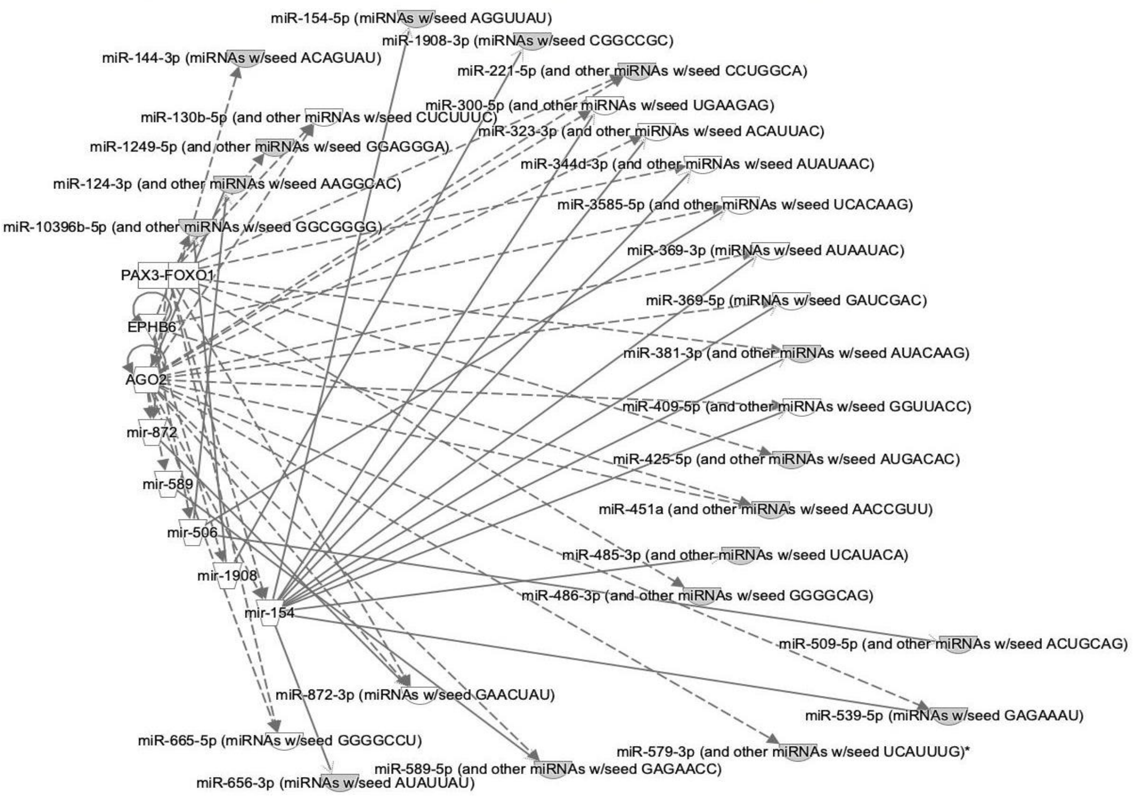

Network 2:

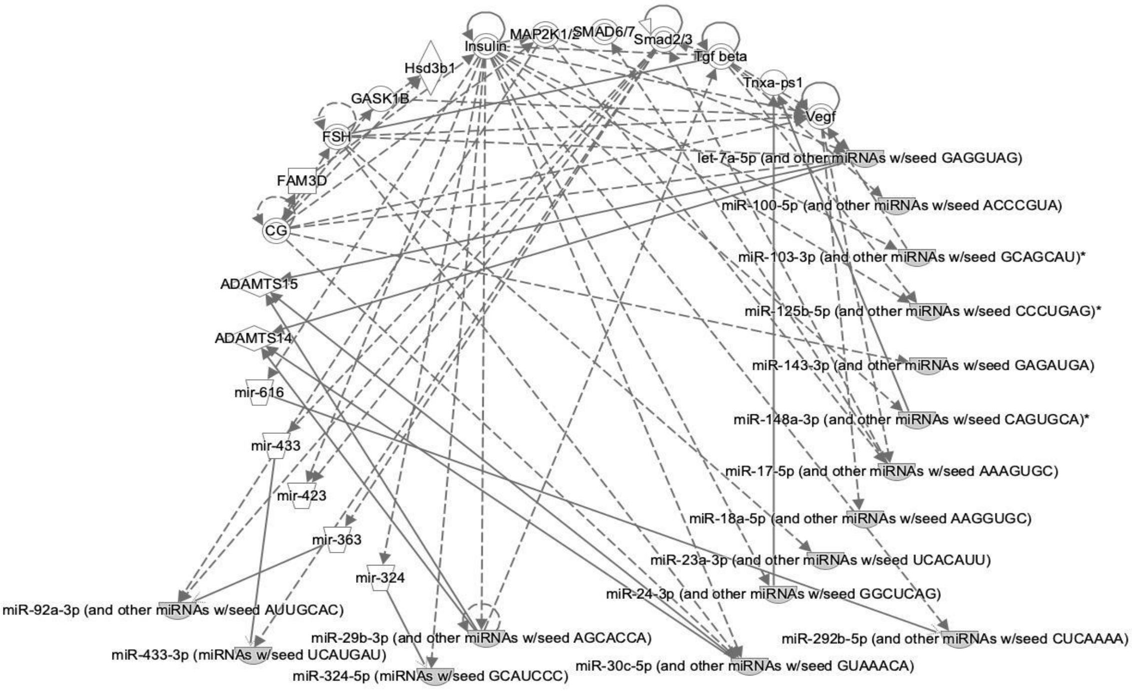


Figure S9: Ingenuity Pathway (IPA) functional analysis to generate networks of diseases and disorders associated with miRNAs. Two networks with the highest scores are illustrated. Network 1 (score:33) included functions associated with organismal injury and abnormalities, skeletal and muscular system development and function, and tissue morphology, and associated with AGO2, EPHB6, PAX3-FOXO1. Network 2 (score: 31) included glomerular injury, Inflammatory disease, and inflammatory response, and was associated with ADAMTS14, ADAMTS15, CG,

FAM3D, FSH, GASK1B, Hsd3b1, INSULIN, MAP2K1, SMAD6/7, TGF-beta, Tmxa-ps1, and VEGF.

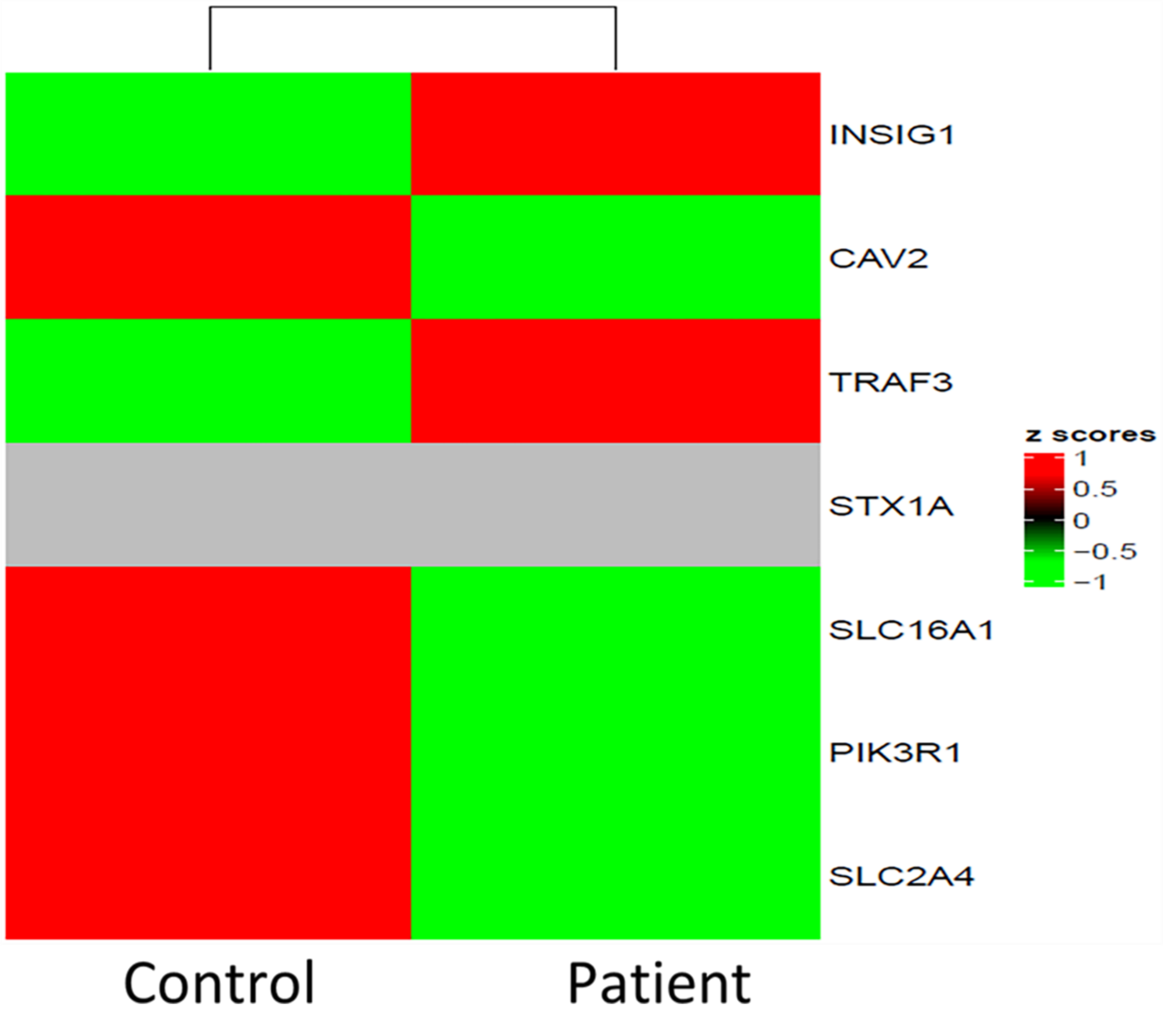


Figure S10: miR-29a-3b overexpression in the patient carrying the intronic GLUT2 mutation coincides with the downregulation of genes involved in insulin production and secretion. CAV2, SLC16A1, PIK3R1, and SLC2A4 were found to be downregulated in the patient in comparison to the healthy control.

a-1)

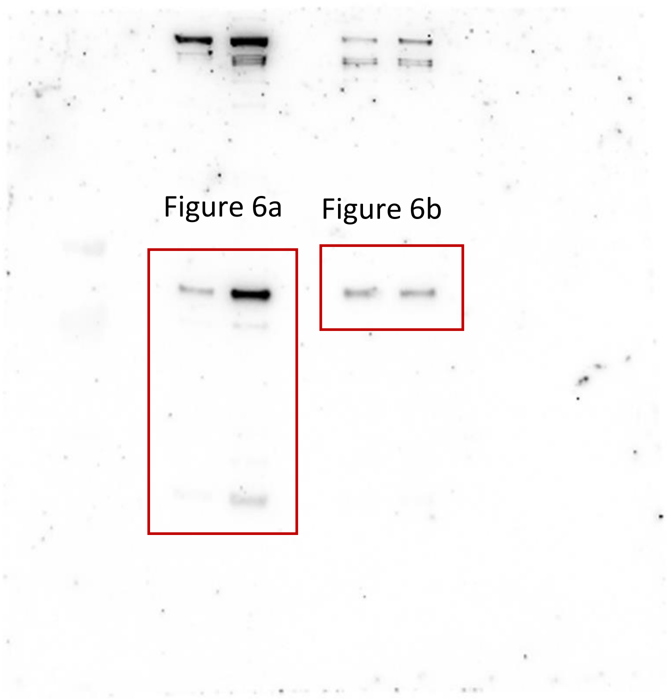

a-2)

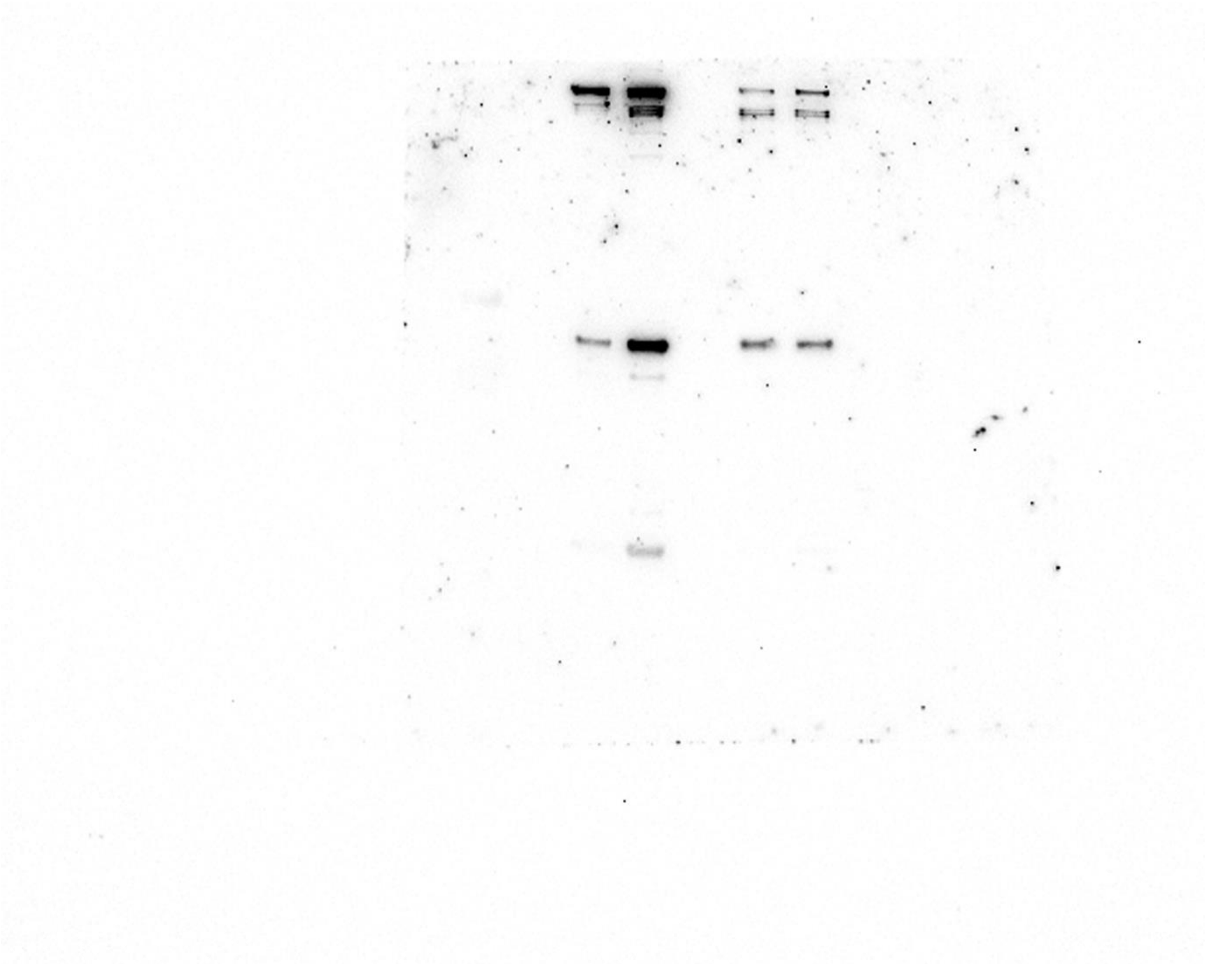

a-3)

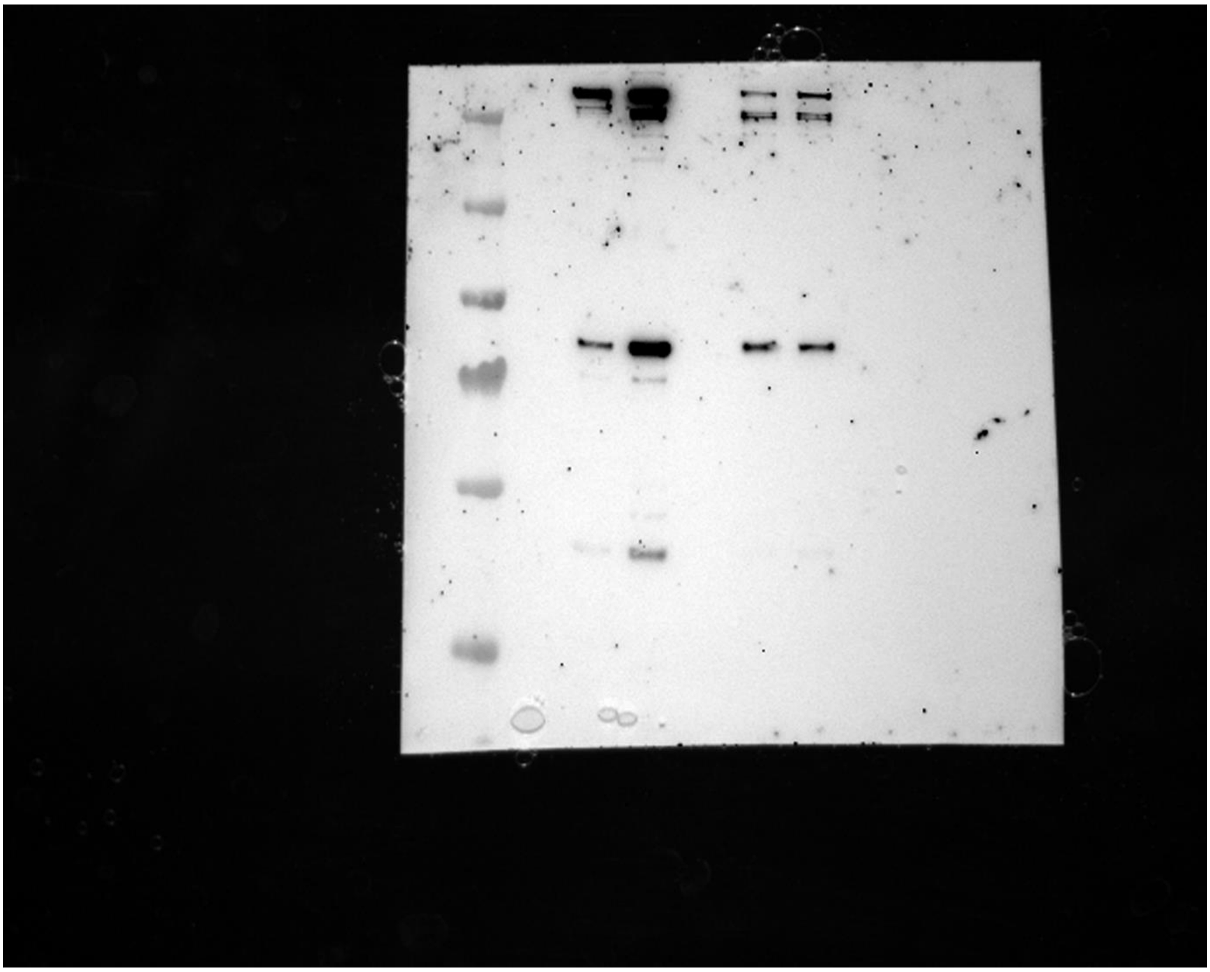

b-1)

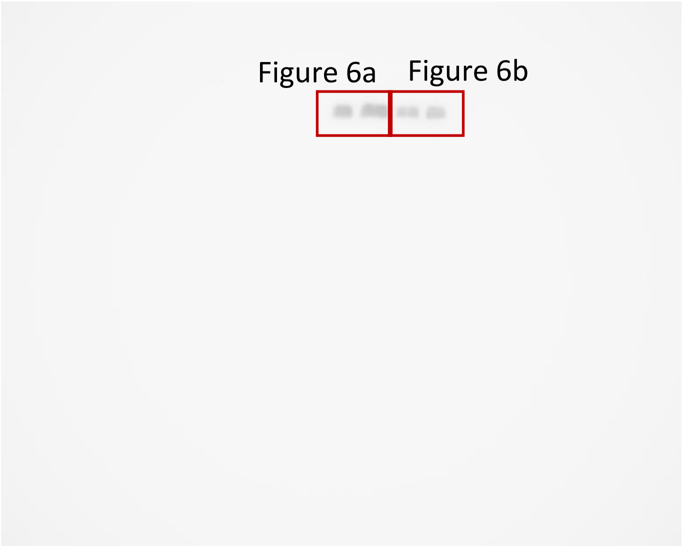


b-2)

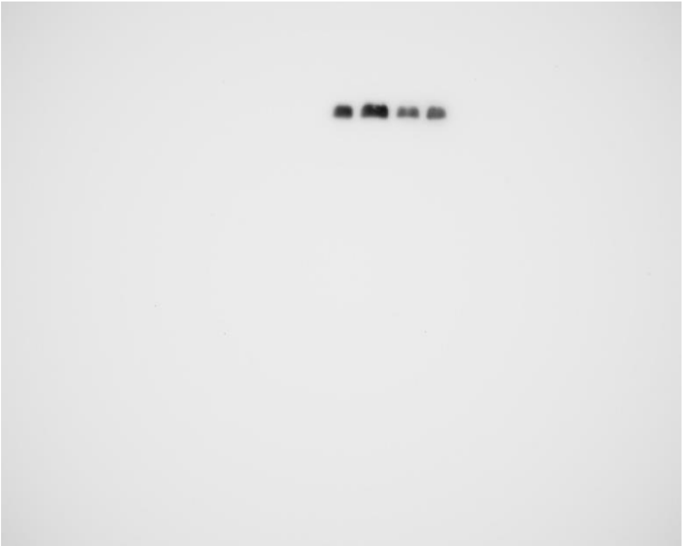

Figure S11: Full-length images of western blots (Figure 6). a 1-3 are multiple exposures using the GLUT2 antibody. b 1-2 are multiple exposures using beta-actin antibodies. The red parts in a.1 and b.1 are the cropped parts shown in Figure 6.
